# Risk Factors of Language Delay at Two Years of Corrected Age among Very-Low-Birth-Weight Preterm Infants: A Population-Based Study

**DOI:** 10.3390/children10020189

**Published:** 2023-01-19

**Authors:** Wei-Lun Tseng, Chia-Huei Chen, Jui-Hsing Chang, Chun-Chih Peng, Wai-Tim Jim, Chia-Ying Lin, Chyong-Hsin Hsu, Tzu-Yu Liu, Hung-Yang Chang, on behalf of the Taiwan Premature Infant Follow-up Network

**Affiliations:** 1 Department of Pediatrics, MacKay Children’s Hospital, Taipei 104217, Taiwan; 2 Department of Medicine, MacKay Medical College, New Taipei City 251020, Taiwan; 3 Department of Pediatrics, Hsinchu MacKay Memorial Hospital, Hsinchu City 30046, Taiwan; 4 Premature Baby Foundation of Taiwan, Taipei 10491, Taiwan

**Keywords:** preterm infants, very low birth weight, language development

## Abstract

Language delays are often underestimated in very-low-birth-weight (VLBW) preterm infants. We aimed to identify the risk factors of language delay at two years of corrected age in this vulnerable population. VLBW infants, who were assessed at two years of corrected age using the Bayley Scale of Infant Development, third edition, were included using a population-based cohort database. Language delay was defined as mild to moderate if the composite score was between 70 and 85 and severe if the score was < 70. Multivariable logistic regression analysis was used to identify the perinatal risk factors associated with language delay. The study comprised 3797 VLBW preterm infants; 678 (18%) had a mild to moderate delay and 235 (6%) had a severe delay. After adjusting for confounding factors, low maternal education level, low maternal socioeconomic status, extremely low birth weight, male sex, and severe intraventricular hemorrhage (IVH) and/or cystic periventricular leukomalacia (PVL) were found to be significantly associated with both mild to moderate and severe delays. Resuscitation at delivery, necrotizing enterocolitis, and patent ductus arteriosus requiring ligation showed significant associations with severe delay. The strongest factors predicting both mild to moderate and severe language delays were the male sex and severe IVH and/or cystic PVL; thus, early targeted intervention is warranted in these populations.

## 1. Introduction

Perinatal and neonatal care has progressed significantly in the last few decades, with marked improvements in the survival of very-low-birth-weight (VLBW, birth weight (BW) ≤ 1500 g) infants [1,2,3]. However, the challenges of VLBW infants include not only survival but also short-term neonatal morbidity and long-term neurodevelopmental outcomes [4,5]. Studies on neurodevelopmental outcomes in VLBW infants have mainly focused on cognitive or motor development. Language is less mentioned but language impairment is crucial for social, emotional, and behavioral functions and even affects employment opportunities later in life [6,7].

Several perinatal risk factors have been reported to be related to language delay, including higher maternal age [8], lower maternal education level [9,10,11,12,13,14,15], preterm birth [11,13,14,16,17,18,19], BW < 1000 g, VLBW [11,20,21], male sex [13,14,15,17], bronchopulmonary dysplasia (BPD) [11,22], and periventricular leukomalacia (PVL) [23]. Preterm birth has been reported to be a risk factor for language delay in many studies, and some studies have focused on this group to determine the factors that predominantly affect language development among preterm infants. However, these risk factors were not consistent in all studies and require further investigation [9,11,13,15,17,20,21,23,24]. This study aimed to assess the risk factors of language delay in VLBW preterm infants at 24 months of corrected age (CA). By identifying high-risk infants, intervention programs may be initiated to prevent further delays earlier, thereby compensating for deficits and promoting optimal function and independence.

## 2. Materials and Methods

### 2.1. Participants

This retrospective study was approved by the Mackay Memorial Hospital Institutional Review Board (approval no.: 20MMHIS040e). We evaluated VLBW infants who were admitted to any of the 22 member hospitals of the Taiwan Premature Infant Follow-up Network between January 2010 and December 2015 and who had a complete Bayley Scale of Infant Development, third edition (BSID-III), at a CA of 24 months. This network was funded by the Premature Baby Foundation of Taiwan.

All infants underwent screening for hearing function before discharge and those with severe hearing impairment, requiring a hearing aid, were excluded. Term infants or VLBW infants with major chromosomal or structural anomalies, not using BSID-III for follow-up, and those who died or were lost to follow-up before a CA of 24 months were also excluded.

The data on intrapartum and demographic variables were collected and compared between the groups. A low maternal education level was defined as a mother receiving education for <12 years because Taiwan has compulsory education for up to 12 years. Maternal socioeconomic status, determined by education and occupation, was categorized into five classes (I–V), with class I representing the highest status and class V the lowest [25]. IUGR was diagnosed by obstetricians for fetus with an estimated weight of fetus below the 10th percentile for gestational age. Resuscitation at delivery was defined as the requirement for positive pressure ventilation, intubation, or chest compression. BPD was defined according to the criteria of the National Institute of Child Health and Human Development criteria [26]. Severe intraventricular hemorrhage (IVH) was defined as grade III or IV, according to the criteria described by Papile et al. [27]. Necrotizing enterocolitis (NEC) was defined as stage II or III according to the modified Bell’s staging criteria [28]. Retinopathy of prematurity (ROP) that needed treatment was defined as patients receiving laser or bevacizumab injection treatment.

### 2.2. Outcome Evaluation

At 24 months CA, the infants were evaluated by a certified psychologist using BSID-III. The BSID-III has been widely used for the early identification of developmental delay and the need for early intervention services in high-risk infants. It comprises three individual developmental scores: cognitive, language, and motor composite scores. In this study, we focused on the language composite score generated by receptive and expressive communication. It assesses communication skills by using language and gestures. The composite scores of the BSID-III were age-standardized norm-referenced scores, with a mean score of 100 and a standard deviation (SD) of 15. Using the language composite score of the BSID-III, outcomes were classified as normal (≥85), mild to moderate (70–85 (<−1 SD)), or severe (<70 (<−2 SD)) language delay.

### 2.3. Statistical Analysis

We compared intrapartum and demographic variables between normal, mild to moderate, and severe language delay groups to identify risk factors. Categorical data were analyzed using the chi-squared test and continuous data were analyzed using an independent t-test. The risk factors in previous analyses were used in the logistic regression model to determine the real significant risk factors of both mild to moderate and severe language delay groups. These risk factors were also analyzed to determine their relationship with receptive and expressive communication raw scores in a linear regression model. Odds ratios (ORs) and 95% confidence intervals (CIs) were calculated for all outcomes. Pearson’s correlation was used to test the correlation between gestational age (GA), BW, and language composite score.

All statistical analyses were performed using IBM SPSS Statistics for Windows version 25.0 (IBM Corp., Armonk, NY, USA). Differences were considered statistically significant at *p* < 0.05.

## 3. Results

### 3.1. Participant Selection and Recruitment Period

**Figure 1:**
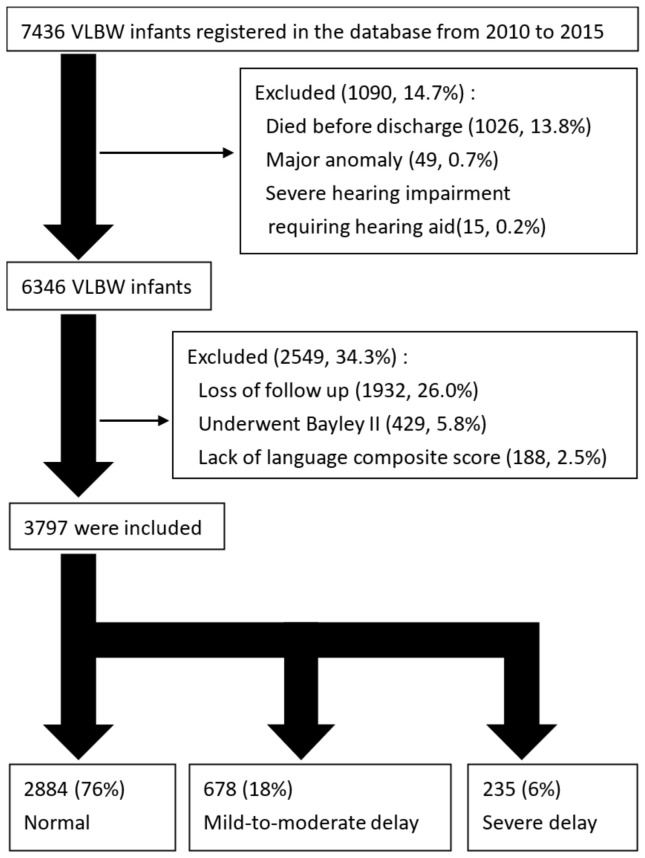
Flowchart of the study populations.

Figure 1 illustrates the database enrollment process. Between 2010 and 2015, 7436 live-born preterm infants were registered in the network; 1090 infants were excluded because of death before discharge, major congenital anomalies, or severe hearing impairment requiring a hearing aid at a CA of 24 months. Among the remaining 6346 infants, 2549 were excluded because of loss to follow-up after discharge, lack of language composite score, or evaluation with BSID-II at a CA of 24 months. Thus, 3797 VLBW infants were included in the final analyses and divided into three groups—normal (*n* = 2884, 76% of participants), mild to moderate language delay (*n* = 678, 18% of participants), and severe language delay (*n* = 235, 6% of participants).

### 3.2. Risk Factors of Mild to Moderate or Severe Language Delay

Factors associated with both mild to moderate and severe language delay are shown in Table 1, including lower maternal education level, lower maternal socioeconomic status, GA < 28 weeks, BW < 1000 g, male sex, resuscitation at delivery, respiratory distress syndrome (RDS) requiring surfactant, mechanical ventilation (MV), patent ductus arteriosus (PDA) needing ligation, BPD, ROP needing treatment, and severe IVH and/or PVL. Sepsis and NEC were associated with severe language delay but not with mild to moderate delay.

### 3.3. Logistic Regression Model for Risk Factors

The factors that showed a significant relation with both mild to moderate delay and severe delay, described in Section 3.2, were subjected to logistic regression analysis separately in both groups. The logistic regression models were both statistically significant; χ2(12) = 198.488, *p* < 0.001 for severe delay and χ2(10) = 219.124, *p* < 0.001 for mild to moderate delay. The model for severe delay explained 15.0% (Nagelkerke R2) of the variance and correctly classified 92.4% of cases, while that for mild to moderate delay explained 9.7% (Nagelkerke R2) of the variance and correctly classified 81.1% of cases. After logistic regression, the statistically significant risk factors of both mild to moderate and severe language delay were lower maternal socioeconomic status, male sex, and severe IVH and/or PVL (Table 2). NEC was found to be a significant risk factor for severe delay but not mild to moderate delay.

### 3.4. Linear Regression Model for Language Raw Scores

The risk factors of the delayed receptive or expressive use of BSID-III raw scores are presented in Table 3. Except for NEC, which only affected receptive scores, the other factors affected both receptive and expressive language. In the regression model, the most strongly associated factor with poor receptive and expressive language outcomes was severe IVH and/or PVL. NEC was significantly associated with receptive language delay, and the male sex was associated with expressive language delay.

### 3.5. Correlation of Language Delay with GA and BW

We also examined the correlation of language delay with GA and BW, both significantly associated with mild to moderate and severe delays. The Pearson correlation between GA and language composite scores and between BW and language composite scores was *r*(3795) = 0.187, *p* < 0.001, and *r*(3795) = 0.213, *p* < 0.001, respectively. The *p*-values of both groups were < 0.01 but the Pearson correlation coefficients were both less than 0.3, suggesting a statistically significant but low degree of correlation.

## 4. Discussion

This is the largest study that attempted to identify the risk factors of language delay in VLBW preterm infants. Risk factors of both mild to moderate and severe delay were lower maternal education level, lower maternal socioeconomic status, extremely preterm birth, BW < 1000 g, male sex, resuscitation at delivery, PDA undergoing ligation, ROP requiring treatment, BPD, and severe IVH and/or PVL. In contrast, NEC and sepsis were related to severe delay. The strongest predictors of mild to moderate and severe language delays were male sex and IVH and/or PVL, respectively.

In agreement with previous studies on term or preterm infants, male sex, lower maternal education level, and maternal socioeconomic status were reported as significant risk factors for language delay [9,10,11,13,14,15,16]. In our study, these three risk factors had relatively high ORs, suggesting their strong predictive value. A higher maternal education level had been reported to be associated with some protective factors, such as better feeding and hygiene practices and frequent utilization of the antenatal care [29,30]. Mothers with low educational levels may be less sensitive to their infants’ needs, which is associated with malnutrition in children and depression and stress in mothers [31,32,33,34]. Advanced maternal age had been reported to be related to severe language delay [8], but we did not find this relationship in our study. This may be because the previous study used a single-center database and had different exclusion criteria.

Although there are many studies on the relationship between preterm birth, low BW, and language development, the results vary owing to different study designs. Some studies have suggested that preterm birth alone is not a risk factor for language delay [35,36], while others have shown that smaller GA or lower BW is not related to language delay in normal-term infants [15,37]. Our analysis between BW and GA with language composite scores suggested a statistically significant but low-degree correlation; therefore, using a cut-off threshold to separate the two groups might better explain language development. We found that BW < 1000 g and GA < 28 weeks showed significant correlations with mild to moderate and severe delays in the first analysis. However, after logistic regression, only BW < 1000 g was statistically significant. This result cannot be explained by SGA since there was no significant correlation between SGA and mild to moderate or severe delay, which was consistent with the findings of some previous studies [9,11,24]. Thus, our results suggest that BW < 1000 g is a strong risk factor for language delay; however, this requires further investigation.

Preterm birth has been reported to affect brain development, including cortical growth, expansion, folding, and microstructure, in some studies [38,39,40,41,42]. Both severe IVH and PVL have been reported to affect neurodevelopment in preterm infants, especially motor and cognitive development, but their roles in language development are still controversial [23,43,44,45]. In our study, we found that severe IVH and/or PVL were the strongest predictors of language delay. This highlights the importance of gently treating preterm infants to avoid these complications.

The gut–brain axis suggests that bowel injury initiates systemic inflammation along with changes in the gut microbiota, which may potentially affect the developing central nervous system. Recent studies have stated that NEC may affect neurodevelopment outcomes via this pathway [46,47]. We found that NEC was highly related to severe delay, but not mild to moderate delay. The OR was much higher than that of other factors and only slightly lower than that of severe IVH and/or PVL. In addition, NEC was found to only be related to receptive scores in our study, but this may need to be confirmed in further research. Our findings suggest that NEC strongly affects language development outcomes and that NEC may express severe neurodevelopmental delay and require early intervention.

Severe ROP has been shown not only to be associated with a delay in white matter maturation and reduced brain volume [48,49] but also to directly affect language development [50]. Visual impairment has been reported to cause early language and communication difficulties [51], which may be a result of a lack of sufficient response from their mothers and interference with mother–infant interactions [52,53].

The major strength of this study is the large sample size of the nationwide database of preterm infants. This study has several limitations. First, it was a retrospective study, due to which some interactions between factors and the causal relationship between the mediation effect and cognitive function could not be completely recognized. However, language development is strongly related to cognitive function [54,55]. Previous studies have highlighted cognitive weaknesses in both term and preterm infants with language weaknesses or delays [56,57,58]. Further prospective studies are needed to clarify the causal relationship between cognitive impairment and language delays. Second, participants represented only 51.1% of the total study population; however, our study is the largest study on preterm infants’ language development and the results may be useful for early targeted intervention in these vulnerable preterm infants. Third, we discussed maternal education and socioeconomic status rather than primary caregivers since these are the only data available in our database. Currently, some researchers have studied the relationships between infants’ neurodevelopment and fathers’ socioeconomic status, educational level, and fathers’ parenting [59,60] and showed that paternal factors also had correlations with infants’ language development. Hence, future studies should use the main caregiver factors rather than maternal factors.

## 5. Conclusions

We identified several risk factors associated with language developmental delay. The strongest factors predicting both mild to moderate and severe language delays were male sex and severe IVH and/or cystic PVL. This information may provide further inspiration when caring for preterm infants to avoid poor neurodevelopmental outcomes and these risk factors can be used to identify those who need early intervention in language development. Further prospective studies are required to clarify the causal relationships between different factors and language development.

## Figures and Tables

**Table 1:** Comparison of maternal and infant demographic characteristics according to language development outcome at 24 months of corrected age.

	N (%) or Mean (SD)							
	Normal (N = 2884)		Mild to Moderate (N = 678)		*p* Value ^#^	Severe (N = 235)		*p* Value ^#^
Mother age ≥ 35	406	14.1%	113	16.7%	0.09	28	11.9%	0.36
Low maternal education level	760	26.4%	297	43.8%	<0.01 *	107	45.5%	<0.01 *
Low maternal socioeconomic status	855	29.6%	319	47.1%	<0.01 *	122	51.9%	<0.01 *
Preeclampsia	591	20.5%	137	20.2%	0.87	45	19.1%	0.62
PPROM	917	31.8%	215	31.7%	0.97	62	26.4%	0.09
Chorioamnionitis	136	4.7%	22	3.2%	0.09	13	5.5%	0.57
IUGR	332	11.5%	79	11.7%	0.92	33	14.0%	0.25
2 doses of prenatal steroid	1661	57.6%	381	56.2%	0.51	130	55.3%	0.50
GA	29.1	(2.7)	28.3	(2.8)	<0.01 *	27.9	(2.9)	<0.01 *
GA < 28 weeks	813	28.2%	266	39.2%	<0.01 *	118	50.2%	<0.01 *
BW	1135.5	(248.6)	1035.2	(266.5)	<0.01 *	988.5	(289.1)	<0.01 *
BW < 1000 g	864	30.0%	299	44.1%	<0.01 *	124	52.8%	<0.01 *
SGA	957	33.2%	222	32.7%	0.83	75	31.9%	0.69
Sex (male)	1335	46.3%	410	60.5%	<0.01 *	153	65.1%	<0.01 *
Singleton	1953	67.7%	466	68.7%	0.51	160	68.1%	0.91
Resuscitation at delivery	1748	60.6%	473	69.8%	<0.01 *	181	77.0%	<0.01 *
Sepsis	468	16.2%	124	18.3%	0.19	66	28.1%	<0.01 *
NEC	33	1.1%	9	1.3%	0.69	12	5.1%	<0.01 *
PDA ligation	427	14.8%	152	22.4%	<0.01 *	69	29.4%	<0.01 *
ROP needed treatment	225	7.8%	103	15.2%	<0.01 *	46	19.6%	<0.01 *
BPD	1851	64.2%	516	76.1%	<0.01 *	185	78.7%	<0.01 *
Severe IVH and/or PVL	171	5.9%	82	12.1%	<0.01 *	52	22.1%	<0.01 *

Data were expressed as (*n*, %). Abbreviations: PPROM, preterm premature rupture of membrane; IUGR, intrauterine growth restriction; GA, gestational age; BW, birth weight; SGA, small for gestational age; NEC, necrotizing enterocolitis; PDA, patent ductus arteriosus; ROP, retinopathy of prematurity; BPD, bronchopulmonary dysplasia; IVH, intraventricular hemorrhage; PVL, periventricular leukomalacia; * *p* < 0.05; # comparing to normal group.

**Table 2:** Risk factors of mild to moderate and severe language delay.

	Mild To Moderate Delay				Severe Delay			
	Wald χ2	OR	95% CI	*p* Value	Wald χ2	OR	95% CI	*p* Value
Low maternal education level	14.836	1.579	1.251–1.992	< 0.01 *	6.390	1.598	1.111–2.298	0.01 *
Low maternal socioeconomic status	16.481	1.605	1.277–2.017	< 0.01 *	11.673	1.872	1.306–2.681	<0.01 *
GA < 28 weeks	1.086	1.141	0.890–1.463	0.30	0.065	1.052	0.712–1.556	0.80
BW < 1000 g	11.028	1.478	1.174–1.861	<0.01 *	7.774	1.681	1.167–2.421	<0.01 *
Gender (male)	44.904	1.834	1.536–2.190	<0.01 *	27.631	2.181	1.631–2.917	<0.01 *
resuscitation at delivery	3.086	1.197	0.979–1.462	0.08	5.330	1.501	1.063–2.120	0.02 *
Sepsis					2.119	1.292	0.922–1.811	0.14
NEC					7.182	2.763	1.314–5.809	<0.01 *
PDA ligation	3.016	1.236	0.973–1.571	0.08	6.606	1.580	1.115–2.239	0.01 *
ROP needed treatment	6.521	1.456	1.091–1.942	0.01 *	0.979	1.237	0.812–1.885	0.32
BPD	3.049	1.227	0.975–1.544	0.08	0.449	0.873	0.586–1.300	0.50
Severe IVH and/or PVL	13.064	1.728	1.284–2.324	<0.01 *	34.369	3.102	2.125–4.529	<0.01 *

Abbreviations: OR, odds ratios; CI, confidence intervals; GA, gestational age; BW, birth weight; NEC, necrotizing enterocolitis; PDA, patent ductus arteriosus; ROP, retinopathy of prematurity; BPD, bronchopulmonary dysplasia; IVH, intraventricular hemorrhage; PVL, periventricular leukomalacia; * *p* < 0.05.

**Table 3:** Risk factors correlating with receptive and expressive language scores.

	Receptive Raw Score				Expressive Raw Score			
	B	SE	β	*p* Value	B	SE	β	*p* Value
Low maternal education level	−1.40	0.22	−0.13	<0.01 *	−1.43	0.35	−0.08	<0.01 *
Low maternal socioeconomic status	−1.26	0.21	−0.12	<0.01 *	−1.46	0.34	−0.09	<0.01 *
GA < 28 weeks	−0.11	0.23	−0.01	0.64	−0.49	0.37	−0.03	0.18
BW < 1000 g	−0.71	0.21	−0.07	<0.01 *	−0.56	0.34	−0.03	0.10
Sex, (male)	1.32	0.15	0.13	<0.01 *	1.72	0.25	0.11	0.01 *
Resuscitation at delivery	−0.34	0.17	−0.03	0.05 *	−0.07	0.28	0.00	0.81
Sepsis	−0.14	0.21	−0.01	0.50	−0.50	0.34	−0.02	0.14
NEC	−1.75	0.66	−0.04	<0.01 *	−0.26	1.06	0.00	0.81
PDA ligation	−0.19	0.23	−0.01	0.40	−1.28	0.37	−0.06	<0.01 *
ROP needed treatment	−0.68	0.29	−0.04	0.02 *	−1.33	0.46	−0.05	<0.01 *
BPD	0.22	0.19	0.02	0.26	0.31	0.31	0.02	0.32
Severe IVH and/or cystic PVL	−2.07	0.29	−0.11	<0.01 *	−2.55	0.47	−0.09	<0.01 *

Abbreviations: B, unstandardized beta; SE, standard error; β, standardized beta; GA, gestational age; BW, birth weight; NEC, necrotizing enterocolitis; PDA, patent ductus arteriosus; ROP, retinopathy of prematurity; BPD, bronchopulmonary dysplasia; IVH, intraventricular hemorrhage; PVL, periventricular leukomalacia; * *p* < 0.05.

## Data Availability

The data analyzed in this study are subject to the following licenses/restrictions: the data that support the findings of this study are available from the Premature Baby Foundation of Taiwan. Restrictions apply to the availability of these data, which were used under the license for this study. Data are not available without the permission of the Premature Baby Foundation of Taiwan. Requests to access these datasets should be directed to the Premature Baby Foundation of Taiwan (pbf@pbf.org.tw).

## References

[1] Chang J.H., Hsu C.H., Tsou K.I., Jim W.T., Taiwan Premature Infant Developmental Collaborative Study Group (2018). Outcomes and related factors in a cohort of infants born in Taiwan over a period of five years (2007–2011) with borderline viability. J. Formos. Med. Assoc..

[2] Su Y.-Y., Wang S.-H., Chou H.-C., Chen C.-Y., Hsieh W.-S., Tsao P.-N., Tsou K.-I., Hsu C.-H., Mu S.-C., Lin H.-C. (2016). Morbidity and mortality of very low birth weight infants in Taiwan—Changes in 15 years: A population based study. J. Formos. Med. Assoc..

[3] Su B.-H., Hsieh W.-S., Hsu C.-H., Chang J.-H., Lien R., Lin C.-H., Premature Baby Foundation of Taiwan (PBFT) (2015). Neonatal outcomes of extremely preterm infants from Taiwan: Comparison with Canada, Japan, and the USA. Pediatr. Neonatol..

[4] Lin C.-Y., Hsu C.-H., Chang J.-H., Taiwan Premature Infant Follow-up Network (2020). Neurodevelopmental outcomes at 2 and 5 years of age in very-low-birth-weight preterm infants born between 2002 and 2009: A prospective cohort study in Taiwan. Pediatr. Neonatol..

[5] Chen P.-S., Jeng S.-F., Tsou K.-I., Taipei Long-Term Developmental Follow-Up Group for Preterm Infants (2004). Developmental function of very-low-birth-weight infants and full-term infants in early childhood. J. Formos. Med. Assoc..

[6] Conti-Ramsden G., Mok P.L., Pickles A., Durkin K. (2013). Adolescents with a history of specific language impairment (SLI): Strengths and difficulties in social, emotional and behavioral functioning. Res. Dev. Disabil..

[7] Law J., Rush R., Schoon I., Parsons S. (2009). Modeling Developmental Language Difficulties from School Entry into Adulthood: Literacy, Mental Health, and Employment Outcomes. J. Speech Lang. Hear. Res..

[8] Tseng K.-T., Peng C.-C., Chang J.-H., Hsu C.-H., Lin C.-Y., Jim W.-T., Chang H.-Y. (2019). The impact of advanced maternal age on the outcomes of very low birth weight preterm infants. Medicine.

[9] Sania A., Sudfeld C.R., Danaei G., Fink G., McCoy D.C., Zhu Z., Fawzi M.C.S., Akman M., Arifeen S.E., Barros A.J.D. (2019). Early life risk factors of motor, cognitive and language development: A pooled analysis of studies from low/middle-income countries. BMJ Open.

[10] El-Din E.M.S., Elabd M.A., Nassar M.S., Metwally A.M., Abdellatif G.A., Rabah T.M., Shalaan A., Shaaban S.Y., Kandeel W., El Etreby L.A. (2019). The Interaction of Social, Physical and Nutritive Factors in Triggering Early Developmental Language Delay in a Sample of Egyptian Children. Open Access Maced. J. Med. Sci..

[11] Agarwal P.K., Shi L., Rajadurai V.S., Zheng Q., Yang P.H., Khoo P.C., Quek B.H., Daniel L.M. (2018). Factors affecting neurodevelopmental outcome at 2 years in very preterm infants below 1250 grams: A prospective study. J. Perinatol..

[12] Asztalos E.V., Church P.T., Riley P., Fajardo C., Shah P.S., Canadian Neonatal Network and Canadian Neonatal Follow-up Network Investigators (2017). Association between primary caregiver education and cognitive and language development of preterm neonates. Am. J. Perinatol..

[13] Patra K., Greene M.M., Patel A.L., Meier P. (2016). Maternal education level predicts cognitive, language, and motor outcome in preterm infants in the second year of life. Am. J. Perinatol..

[14] Nishimura T., Takei N., Tsuchiya K., Asano R., Mori N. (2016). Identification of neurodevelopmental trajectories in infancy and of risk factors affecting deviant development: A longitudinal birth cohort study. Int. J. Epidemiol..

[15] Korpilahti P., Kaljonen A., Jansson-Verkasalo E. (2016). Identification of biological and environmental risk factors for language delay: The Let’s Talk STEPS study. Infant Behav. Dev..

[16] Do C.H.T., Kruse A.Y., Wills B., Sabanathan S., Clapham H., Pedersen F.K., Pham T.N., Vu P.M., Børresen M.L. (2020). Neurodevelopment at 2 years corrected age among Vietnamese preterm infants. Arch. Dis. Child..

[17] Sanchez K., Spittle A.J., Cheong J.L., Thompson D., Doyle L.W., Anderson P.J., Morgan A.T. (2019). Language in 2-year-old children born preterm and term: A cohort study. Arch. Dis. Child..

[18] Zambrana I.M., Vollrath M.E., Sengpiel V., Jacobsson B., Ystrom E. (2016). Preterm delivery and risk for early language delays: A sibling-control cohort study. Int. J. Epidemiol..

[19] Rechia I.C., Oliveira L.D., Crestani A.H., Biaggio E.P.V., De Souza A.P.R. (2016). Effects of prematurity on language acquisition and auditory maturation: A systematic review. CoDAS.

[20] Dehghan M., Kuhi M., Rezvani S., Esmaeilzadeh S., Samadinezhad H., Basirat Z., Mir F.N., Khafri S., Ahmadi A. (2020). Speech and language development of children born following assisted reproductive technologies. Int. J. Pediatr. Otorhinolaryngol..

[21] Ahn S.H., Kim S.A. (2017). Assessment of preterm infants using the Bayley-III scales in Korea. Ann. Rehabil. Med..

[22] Choi E.K., Shin S.H., Kim E.-K., Kim H.-S. (2019). Developmental outcomes of preterm infants with bronchopulmonary dysplasia-associated pulmonary hypertension at 18–24 months of corrected age. BMC Pediatr..

[23] Youn Y., Moon C.-J., Sung I.K. (2017). Long-term postnatal steroid effect in very low birth weight infants. Steroids.

[24] Ballot D.E., Ramdin T., Rakotsoane D., Agaba F., Chirwa T., Davies V.A., Cooper P.A. (2017). Assessment of developmental outcome in very low birth weight infants in Southern Africa using the Bayley Scales of Infant Development (III). BMJ Paediatr. Open.

[25] Liu T.-Y., Chang J.-H., Peng C.-C., Hsu C.-H., Jim W.-T., Lin J.-Y., Chen C.-H., Li S.-T., Chang H.-Y. (2021). Predictive Validity of the Bayley-III Cognitive Scores at 6 Months for Cognitive Outcomes at 24 Months in Very-Low-Birth-Weight Infants. Front. Pediatr..

[26] Jobe A.H., Bancalari E. (2001). Bronchopulmonary Dysplasia. Am. J. Respir. Crit. Care Med..

[27] Papile L.-A., Burstein J., Burstein R., Koffler H. (1978). Incidence and evolution of subependymal and intraventricular hemorrhage: A study of infants with birth weights less than 1,500 gm. J. Pediatr..

[28] Neu J. (1996). Necrotizing Enterocolitis: The Search for a Unifying Pathogenic Theory Leading to Prevention. Pediatr. Clin. N. Am..

[29] Fein S.B., Labiner-Wolfe J., Scanlon K.S., Grummer-Strawn L.M. (2008). Selected Complementary Feeding Practices and Their Association with Maternal Education. Pediatrics.

[30] Nisar N., White F. (2003). Factors affecting utilization of antenatal care among reproductive age group women (15-49 years) in an urban squatter settlement of Karachi. J. Pak. Med. Assoc..

[31] Makoka D., Masibo P.K. (2015). Is there a threshold level of maternal education sufficient to reduce child undernutrition? Evidence from Malawi, Tanzania and Zimbabwe. BMC Pediatr..

[32] Van Ryzin M.J., Carlson E.A., Sroufe L.A. (2011). Attachment discontinuity in a high-risk sample. Attach. Hum. Dev..

[33] Chittleborough C.R., Lawlor D.A., Lynch J.W. (2011). Young Maternal Age and Poor Child Development: Predictive Validity from a Birth Cohort. Pediatrics.

[34] Jaffari-Bimmel N., Juffer F., van Ijzendoorn M.H., Bakermans-Kranenburg M.J., Mooijaart A. (2006). Social development from infancy to adolescence: Longitudinal and concurrent factors in an adoption sample. Dev. Psychol..

[35] Pérez-Pereira M., Fernández P., Gómez-Taibo M.L., Resches M. (2014). Language development of low risk preterm infants up to the age of 30months. Early Hum. Dev..

[36] Pérez-Pereira M., Fernández P., Resches M., Gómez-Taibo M.L. (2013). Determinants of early language and communication in preterm and full term infants: A comparative study. Enfance.

[37] Madigan S., Wade M.M., Plamondon A., Browne M.D., Jenkins J.M. (2015). Birth Weight Variability and Language Development: Risk, Resilience, and Responsive Parenting. J. Pediatr. Psychol..

[38] Ball G., Seidlitz J., O’Muircheartaigh J., Dimitrova R., Fenchel D., Makropoulos A., Christiaens D., Schuh A., Passerat-Palmbach J., Hutter J. (2020). Cortical morphology at birth reflects spatiotemporal patterns of gene expression in the fetal human brain. PLoS Biol..

[39] Bouyssi-Kobar M., Brossard-Racine M., Jacobs M., Murnick J., Chang T., Limperopoulos C. (2018). Regional microstructural organization of the cerebral cortex is affected by preterm birth. NeuroImage Clin..

[40] Makropoulos A., Aljabar P., Wright R., Hüning B., Merchant N., Arichi T., Tusor N., Hajnal J.V., Edwards A.D., Counsell S.J. (2016). Regional growth and atlasing of the developing human brain. NeuroImage.

[41] Engelhardt E., Inder T.E., Alexopoulos D., Dierker D.L., Hill J., Van Essen D., Neil J.J. (2015). Regional impairments of cortical folding in premature infants. Ann. Neurol..

[42] Ball G., Srinivasan L., Aljabar P., Counsell S.J., Durighel G., Hajnal J.V., Rutherford M.A., Edwards A.D. (2013). Development of cortical microstructure in the preterm human brain. Proc. Natl. Acad. Sci. USA.

[43] Amaral J., Peixoto S., Faria D., Resende C., Taborda A. (2022). Survival and neurodevelopmental outcomes of premature infants with severe peri-intraventricular hemorrhage at 24 months of age. Acta Med. Port..

[44] Cheong J.L.Y., Lee K.J., Boland R.A., Spittle A.J., Opie G.F., Burnett A.C., Hickey L.M., Roberts G., Anderson P.J., Doyle L.W. (2018). Changes in long-term prognosis with increasing postnatal survival and the occurrence of postnatal morbidities in extremely preterm infants offered intensive care: A prospective observational study. Lancet Child Adolesc. Health.

[45] Radic J.A.E., Vincer M., McNeely P.D. (2015). Outcomes of intraventricular hemorrhage and posthemorrhagic hydrocephalus in a population-based cohort of very preterm infants born to residents of Nova Scotia from 1993 to 2010. J. Neurosurg. Pediatr..

[46] Humberg A., Spiegler J., Fortmann M.I., Zemlin M., Marissen J., Swoboda I., Rausch T.K., Herting E., Göpel W., Härtel C. (2020). Surgical necrotizing enterocolitis but not spontaneous intestinal perforation is associated with adverse neurological outcome at school age. Sci. Rep..

[47] Moschopoulos C., Kratimenos P., Koutroulis I., Shah B.V., Möwes A., Bhandari V. (2018). The Neurodevelopmental Perspective of Surgical Necrotizing Enterocolitis: The Role of the Gut-Brain Axis. Mediat. Inflamm..

[48] Sveinsdóttir K., Ley D., Hövel H., Fellman V., Hüppi P.S., Smith L.E., Hellström A., Pupp I.H. (2018). Relation of Retinopathy of Prematurity to Brain Volumes at Term Equivalent Age and Developmental Outcome at 2 Years of Corrected Age in Very Preterm Infants. Neonatology.

[49] Glass T.J.A., Chau V., Gardiner J., Foong J., Vinall J., Zwicker J.G., Grunau R.E., Synnes A., Poskitt K.J., Miller S.P. (2017). Severe retinopathy of prematurity predicts delayed white matter maturation and poorer neurodevelopment. Arch. Dis. Child. Fetal Neonatal Ed..

[50] Arima M., Akiyama M., Fujiwara K., Mori Y., Inoue H., Seki E., Nakama T., Tsukamoto S., Ochiai M., Ohga S. (2020). Neurodevelopmental outcomes following intravitreal bevacizumab injection in Japanese preterm infants with type 1 retinopathy of prematurity. PLoS ONE.

[51] Mosca R., Kritzinger A., van der Linde J. (2015). Language and communication development in preschool children with visual impairment: A systematic review. S. Afr. J. Commun. Disord..

[52] Nagayoshi M., Hirose T., Toju K., Suzuki S., Okamitsu M., Teramoto T., Omori T., Kawamura A., Takeo N. (2017). Related visual impairment to mother-infant interaction and development in infants with bilateral retinoblastoma. Eur. J. Oncol. Nurs..

[53] Nagayoshi M., Hirose T., Omori T., Toju K., Suzuki S., Okamitsu M., Kawamura A., Takeo N. (2015). A Prospective Study of Factors Related to Mother-Infant Interaction in One-year-old Infants with Retinoblastoma. J. Med. Dent. Sci..

[54] Schneider J., Miller S.P. (2019). Preterm brain Injury: White matter injury. Handb. Clin. Neurol..

[55] Twilhaar E.S., Wade R.M., de Kieviet J.F., van Goudoever J.B., van Elburg R.M., Oosterlaan J. (2018). Cognitive Outcomes of Children Born Extremely or Very preterm since the 1990s and Associated Risk Factors: A Meta-analysis and Meta-regression. JAMA Pediatr..

[56] Loeb D.F., Imgrund C.M., Lee J., Barlow S.M. (2020). Language, motor, and cognitive outcomes of toddlers who were born preterm. Am. J. Speech Lang. Pathol..

[57] Bello A., Onofrio D., Remi L., Caselli C. (2018). Prediction and persistence of late talking: A study of Italian toddlers at 29 and 34 months. Res. Dev. Disabil..

[58] Sansavini A., Pentimonti J., Justice L., Guarini A., Savini S., Alessandroni R., Faldella G. (2014). Language, motor and cognitive development of extremely preterm children: Modeling individual growth trajectories over the first three years of life. J. Commun. Disord..

[59] McMahon G.E., Spencer-Smith M., Pace C.C., Spittle A.J., Stedall P., Richardson K., Cheong J.L., Doyle L.W., Anderson P.J., Treyvaud K. (2019). Influence of Fathers’ Early Parenting on the Development of Children Born Very Preterm and Full Term. J. Pediatr..

[60] Pancsofar N., Vernon-Feagans L. (2010). Fathers’ early contributions to children’s language development in families from low-income rural communities. Early Child. Res. Q..

